# Supplementary material for: Improving Psychological Comfort of Paramedics for Field Termination of Resuscitation through Structured Training
Source: Int J Environ Res Public Health. 2021 Jan 25;18(3):1050. doi: 10.3390/ijerph18031050 (PMC7908355; doi:10.3390/ijerph18031050)
Supplement: Supplementary file 1 [file ijerph-18-01050-s001.pdf]

Supplementary Materials

Supplementary table: Regression analysis of change in PCT percentage based on participant characteristics

	Co-efficient	Standard error	P value	95% confidence Interval
**Age**	0.89556	0.4477127	0.049	0.0049168, 1.786203
**Religion**	-4.436751	3.372735	0.192	-11.1462, 2.272692
**Gender**	-0.520984	2.450524	0.832	-5.395857, 4.353889
**Employer (Government)**	1.159396	2.450524	0.730	-5.492993, 7.811785
**Experience as paramedic in years**	-0.8569794	0.4820578	0.079	-1.815946, 0.1019871
**Experience with death and dying**	1.480784	3.045675	0.628	-4.578033, 7.539602
**Number of OHCA treated in the past year**				
**1-5**	0.3438545	5.981527	0.954	-11.55531, 12.24302
**6-10**	0.0399858	6.220648	0.995	-12.33486, 12.41484
**>11**	1.724164	6.362613	0.787	-10.9331, 14.38143
**Number of death pronouncement in the past year**				
**1-5**	11.90268	7.497457	0.116	-3.012154, 26.8175
**6-10**	11.7995	8.071385	0.148	-4.257052, 27.85606
**>11**	7.330513	8.294646	0.379	-9.170181, 23.83121
